# Swallowing function after acute ischemic stroke: Development and validation of a novel clinical prognostic model

**DOI:** 10.3389/fnut.2022.970253

**Published:** 2022-10-05

**Authors:** Peiliang Zhang, Wenbo Zhang, Wujie Shi, Jianbin Weng, Zhongyuan Zhang, Chao Lin, Ning Wang, Zhipeng Shen, Zhi-Lin Chen

**Affiliations:** ^1^Department of Neurosurgery, The Children’s Hospital of Zhejiang University School of Medicine, National Clinical Research Center for Child Health, Hangzhou, China; ^2^Department of Neurology, Translational Research Institute of Brain and Brain-Like Intelligence, Shanghai Fourth People’s Hospital Affiliated to Tongji University School of Medicine, Shanghai, China

**Keywords:** swallowing function, dysphagia, acute ischemic stroke, nasogastric tube, stroke associated pneumonia

## Abstract

**Background:**

Predicting the duration of dysphagia after acute ischemic stroke (AIS) is important for clinical treatment decisions.

**Objective:**

The purpose of this study is to assess the swallowing function of AIS patients and to develop and validate a prognostic model for the need for nasogastric tube (NGT) in these patients.

**Materials and methods:**

We included 554 AIS patients during 2018–2019 as the development group and had 186 AIS patients as the external validation group. The primary end point of the study was the retention of NGT in patients 1 week after admission (Functional Oral Intake Scale ≤ 4). Swallowing function and stroke-associated pneumonia (SAP) at 1 month post-onset were also the objectives of this study. The volume-viscosity swallow test (V-VST) was used to assess the patient’s impaired swallowing function. The Predictive model was built by logistic regression.

**Results:**

Overall, a total of 104 patients required indwelling NGT at 1 week of AIS onset in development group. The final prognostic model includes 5 variables: age (OR: 1.085, 95%CI: 1.049–1.123), neutrophil-to-lymphocyte ratio (NLR) (OR: 1.332, 95%CI: 1.090–1.626), NIHSS (OR: 1.092, 95%CI: 1.025–1.164), history of drinking (OR: 2.532, 95%CI: 1.452–4.417) and stroke location (Subtentorial vs. Supratentorial, OR: 1.954, 95%CI: 1.088–3.509). The prediction model had an AUC of 0.810, while the external validation group was 0.794.

**Conclusion:**

In stroke patients, it is very important to decide early whether to indwell a NGT. The nomogram will support decision making for NGT insertion and help these patients recover from their condition.

## Introduction

Dysphagia affects about half of the estimated number of stroke patients worldwide each year (1, 2). It is one of the common complications of acute ischemic stroke (AIS), and it is associated with pneumonia, prolonged hospitalization, increased mortality, and poor long-term prognosis (2). Dysphagia can lead to inadequate oral intake, causing malnutrition, dehydration, and stroke-associated pneumonia (SAP) (3). In particular, SAP, a typical complication of dysphagia, increases the overall mortality of stroke patients (4, 5). Malnutrition may impair immunity, wound healing, muscle strength and psychological drive. Therefore, hospitals should aim to provide at least adequate nutrition to all patients. Some of these patients will benefit from oral supplementation, but others will require aggressive nutritional support. This can usually be provided through a nasogastric tube (NGT). Clinicians need to decide within the first 48 h whether an NGT should be inserted to prevent these complications (6). However, not all patients who have suffered a stroke will benefit from NGT. Patients with retained NGT often experience nasopharyngeal discomfort, and many have oral pain, thirst, difficulty swallowing, and hoarseness. Local pressure effects from NGT may lead to sinusitis, otitis media, nasal erosion and abscess formation. Short-term damage to the esophagus may include esophagitis, localized abrasions and ulcers due to gastroesophageal reflux, while long-term damage includes significant esophageal strictures (7).

The guidelines recommend feeding with NGT if oral intake is unlikely to resume within 1 week (7). Therefore, clinicians need to predict the duration of impaired swallowing so that NGT can be placed at the appropriate time. There is currently a great deal of variation in the decision to use NGT, which often depends on the subjective experience of the clinician. Nutritional support should be administered as early as possible (within 72 h after stroke), with an emphasis on early and accurate prediction of patients who will require NGT within 1 week (8).

Some studies have explored risk factors associated with long-term impaired swallowing function, including the National Institutes of Health Stroke Scale (NIHSS), SAP, and age.

Although many studies have investigated the factors influencing swallowing function in patients with AIS, it is clear that not just a single factor is involved in this process. Nomogram is a widely used tool that combines multiple predictors to predict patient outcomes. The purpose of this study is to integrate different influencing factors to predict dysphagia in patients with AIS and to give a clinical decision on the need for NGT.

## Materials and methods

### Patients

Between 2018 and 2019, 554 patients with AIS were included in the study as a development group. Only AIS patients who did not require immediate tracheal intubation or neurosurgical intervention were eligible for enrollment. Patients with pre-existing dysphagia or any concomitant disease that could cause dysphagia were excluded. Exclusion criteria: (1) late admission (>48 h after AIS); (2) late initial swallowing assessment (>48 h after AIS); (3) patients lost to follow-up; (4) initial swallowing assessment more than 1 day after admission; (5) Patients with prior dysphagia or stroke recurrence; (6) Patients with pre-existing chronic pneumonia or active infection or fever within 2 weeks prior to admission; (7) history of blood disorders, severe liver disease, cancer or immunosuppressive therapy; (8) Patients with significant decline in consciousness after stroke.

### Dysphagia assessment

The patients meeting the eligibility criteria finished the volume-viscosity swallow test (V-VST) within 48 h of admission. As previously reported, the intention of the V-VST was to determine the impaired efficacy and safety during swallowing (9). Impaired efficacy was defined as labial seal and oral residue, while impaired safety was defined as cough and a ≥3% decrease in oxygen saturation. The last volume that is safe and effective to swallow at different viscosities will be recorded. Based on the results of the V-VST test, the patient’s swallowing function was recorded as 4 grades: Grade I: no dysphagia (safety and efficacy); Grade II: mild dysphagia (safety impaired only); Grade III: moderate dysphagia (efficacy impaired only); Grade IV: severe dysphagia (impaired safety and efficacy).

We use Functional Oral Intake Scale (FOIS) to measure oral intake. FOIS ranges from level 1 to level 7 (from cannot eat through mouth to completely unrestricted oral diet). Severe oral intake deficiency was defined as a single diet or worse (FOIS score ≤ 4), as previous studies have shown a 40% reduction in energy and protein intake and an overall inadequate intake in these individuals, and these patients will be remained NGT.

The primary endpoint was persistent severe oral intake impairment (FOIS score ≤ 4) at 1 week of follow-up, which was based on the following guideline: if oral intake impairment persisted for 1 week, NGT feeding was recommended to be initiated. The secondary endpoint was the retention of NGT in patients 1 month after admission (if oral intake was still expected to be impaired 1 month after stroke, it was recommended for percutaneous endoscopic gastrostomy feeding). The third endpoint is the presence of SAP after AIS.

### Stroke-associated pneumonia diagnostic criteria

SAP in the first week after stroke onset was diagnosed retrospectively on the basis of the Centers for Disease Control and Prevention’s modified criteria: “at least one of the former and one of the latter criteria met: (A) abnormal respiratory examination, pulmonary infiltrates on chest x-rays; (B) productive cough with purulent sputum, microbiological cultures from lower respiratory tract or blood cultures, leukocytosis, elevated CRP” (10).

### Clinical and laboratory assessments

Laboratory examination were assessed on an empty stomach the next morning. Computed tomography (CT) examinations were completed within 24 h of admission. Repeat CT and magnetic resonance imaging (MRI) were performed prior to discharge, and stroke laterality, stroke location, affected arterial territory and number of focus are classified according to MRI. Classification of cerebrovascular events were classified according to the Trial of Org 10,172 in Acute Stroke Treatment (TOAST) criteria (11).

### Development of the statistical model

Baseline patient data and risk factors were statistically analyzed using SPSS. Univariate analysis was performed using chi-square test, *t*-test or Kruskal-Wallis rank sum test. Multivariate analysis was performed using logistic regression analysis. The nomogram is analyzed and constructed by using the R, rms package. After calculating risk factors using logistic regression analysis, we used *P*-values and effect values to construct nomogram and assessed the performance of the nomogram by calculating area under curve (AUC).

### Validation of the statistical model

The prediction accuracy of the model was evaluated by 186 AIS patients as external validation. The receiver operating characteristic (ROC) curve was used to evaluate the accuracy of the model. Calibration plots were used to assess the agreement between predicted and practical results.

## Results

Baseline data for patients in the development group are shown in Table 1. Of the 554 patients in the development group, 104 were indwelt with an NGT within 1 week of onset, with a mean age of 65.01 ± 7.97 years, including 72 males and 32 females, and a mean HIHSS score of 5.26 ± 3.25. The number of patients with good swallowing function who did not require indwelling NGT was 450, their mean age was 59.03 ± 10.68, mean NIHSS score was 3.79 ± 4.18, of which 288 were male and 162 were female.

**TABLE 1:** Clinical baseline characteristics of the development group.

	non-NGT	NGT	P
Age	59.03 ± 10.68	65.01 ± 7.97	<0.001*
BMI	24.58 ± 6.63	23.84 ± 3.39	0.301
SBP	154.30 ± 22.79	162.96 ± 22.10	0.001*
DBP	82.89 ± 13.13	86.25 ± 11.54	0.017*
TB	13.51 ± 11.61	14.96 ± 16.82	0.296
ALT	21.86 ± 13.53	19.03 ± 10.86	0.025*
AST	23.43 ± 12.20	24.54 ± 12.51	0.410
CR	5.09 ± 1.44	5.41 ± 1.77	0.081
Bun	73.09 ± 20.24	75.16 ± 20.30	0.349
HbA1c	6.38 ± 1.55	6.66 ± 1.74	0.121
TC	4.74 ± 1.13	5.01 ± 1.08	0.027*
TG	1.75 ± 1.13	1.63 ± 0.63	0.154
HDL	1.13 ± 0.30	1.19 ± 0.31	0.064
LDL	2.76 ± 0.93	3.04 ± 0.82	0.004*
CRP	6.14 ± 13.33	11.54 ± 20.21	0.013*
NLR	2.36 ± 1.30	3.91 ± 2.54	<0.001*
WBC	6.62 ± 1.80	7.83 ± 2.16	<0.001*
RBC	4.50 ± 0.53	4.52 ± 0.51	0.781
HB	137.15 ± 16.96	136.83 ± 14.30	0.860
PLT	206.42 ± 58.27	213.46 ± 61.70	0.271
NIHSS	3.79 ± 4.18	5.26 ± 3.25	0.001*
Gender (male)	64.00%	69.20%	0.314
Coronary heart disease	5.60%	8.70%	0.235
Hyperlipidemia	5.30%	6.70%	0.576
Hypertension	42.00%	62.50%	0.155
DM	23.10%	23.10%	0.994
Toast (LAA)	54.90%	46.20%	0.108
Stroke laterality			0.859
Left	32.30%	30.30%	
Right	38.30%	37.40%	
Bilateral	29.60%	32.30%	
Stroke location			0.002*
Supratentorial	78.00%	62.50%	
Subtentorial	18.70%	34.60%	
Both	3.30%	2.90%	
Affected arterial territory			0.420
Anterior cerebral	11.60%	9.60%	
Middle cerebral	13.60%	15.40%	
Posterior cerebral	30.70%	30.80%	
Vertebral	5.60%	8.70%	
Basilar	4.90%	1.00%	
Multiple	33.80%	34.60%	
Number of focus (single)	45.30%	37.00%	0.379
Somke	51.10%	46.20%	0.362
Drink	42.00%	62.50%	<0.001*

BMI, Body Mass Index; SBP, Systolic blood pressure; DBP, Diastolic blood pressure; TB, Total bilirubin; ALT, Alanine aminotransferase; AST, Aspartate aminotransferase; BUN, Blood urea nitrogen; Cr, Creatinine; TC, Total cholesterol; TG, Triglyceride; HDL, High density lipoprotein; LDL, Low density lipoprotein; CRP, C-reactive protein; NLR, Neutrophil to lymphocyte ratio; WBC, White blood cell; RBC, Red blood cell; HB, Hemoglobin; PLT, Platelet; NIHSS, National Institutes of Health Stroke Scale on admission; DM, Diabetes mellitus.

*P < 0.05.

**FIGURE 1:**
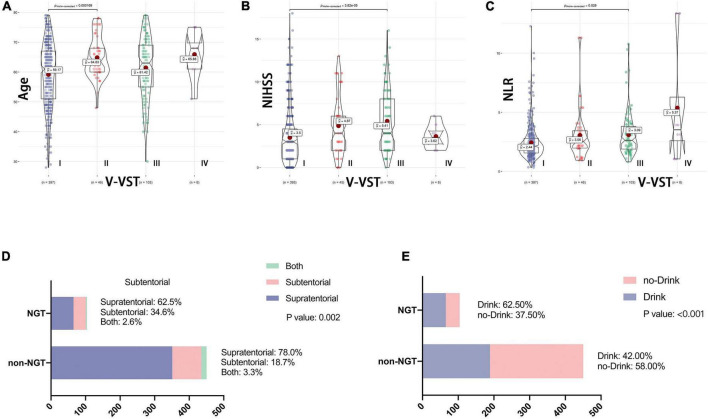
Association between baseline data and swallowing function. V-VST, the volume-viscosity swallow test; NIHSS, National Institutes of Health Stroke Scale on admission; NLR, Neutrophil to lymphocyte ratio; NGT, need for nasogastric tube. Figure shows the correlation analysis between the five variables which were ultimately included in the nomogram and swallowing function. **(A–C)** Shows the relationship between continuous variables including age, NLR and NIHSS and V-VST, all with statistically significant differences in their V-VST grade distributions. **(D,E)** Shows the association between categorical variables including stroke location and drinking and NGT, again with statistical differences.

The V-VST is used to assess the patient’s swallowing function. In univariate analysis, there were significant differences between the two groups (1 week) with respect to five variables: age, NIHSS, neutrophil-to-lymphocyte ratio (NLR), stroke location and history of drinking (Figure 1). The chi-square test showed that the probability of indwelling NGT was higher in alcohol drinkers, while subtentorial lesions alone tended to have indwelling NGT than supratentorial lesions alone (Figure 1).

**FIGURE 2:**
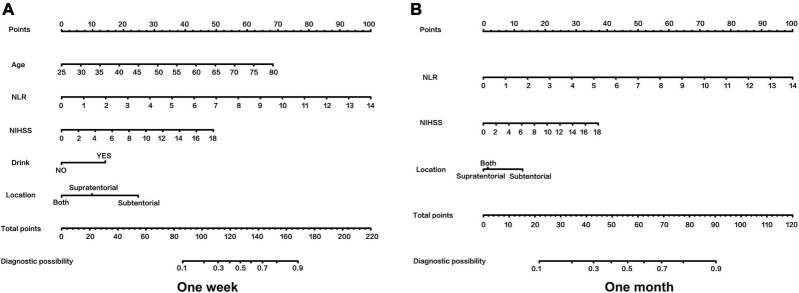
**(A,B)** The nomograms for predicting the prognosis of AIS patients. NIHSS, National Institutes of Health Stroke Scale on admission; NLR, Neutrophil to lymphocyte ratio. To use the nomogram, an individual patient’s value is located on each variable axis, and a line is drawn upward to determine the number of points received for each variable value. The sum of these numbers is located on the Total Points axis, and a line is drawn downward to the probability axis to determine the likelihood of NGT.

The primary endpoint of this study was the indwelling NGT of patients 1 week after admission (FOIS ≤ 4). We used univariate and multivariate analysis to identify potential prognostic factors for NGT indwelling. In univariate analysis, there were significant differences in age (59.03 vs. 65.01, *P* < 0.001), NIHSS (3.79 vs. 5.26, *P* = 0.001), NLR (2.36 vs. 3.91, *P* < 0.001), stroke location (18.7 vs. 34.60%, *P* = 0.002) and drinking history (42.0 vs. 62.50%) between the two groups (1 week) (Table 1). On the basis of univariate analysis, multivariate logistic regression analysis was used to analyze the variables with statistical differences (Table 2). Finally, five variables had independent and significant effects on whether to retain NGT: age (OR: 1.085, 95% CI: 1.049–1.123, *P* < 0.001), NLR (OR: 1.332, 95% CI: 1.090–1.626, P: 0.005), NIHSS (OR: 1.092, 95% CI: 1.025–1.164, P: 0.007), drinking history (OR: 2.532, 95% CI: 1.452–4.417, P: 0.001) and lesion location (subtentorial vs. suprarenal, OR: 1.954, 95% CI: 1.088–3.509, P: 0.025). Based on this, we constructed the corresponding nomogram (Figure 2).

**TABLE 2:** Univariate analysis for the potential factors associated with indwelling nasogastric tube by logistic regression.

	1 week			1 month		
	OR	95% CI	*P*	OR	95% I	*P*
Age	1.085	1.049–1.123	<0.001	1.026	0.989–1.064	0.174
CRP	0.999	0.979–1.020	0.960	–	–	–
NLR	1.332	1.090–1.626	0.005	1.360	1.189–1.555	<0.001*
NIHSS	1.092	1.025–1.164	0.007	1.083	1.018–1.152	0.012*
SBP	0.996	0.981–1.010	0.560	–	–	–
DBP	1.040	0.975–1.022	0.072	–	–	–
ALT	0.998	0.975–1.022	0.862	–	–	–
TC	0.808	0.452–1.444	0.471	–	–	–
LDL	1.735	0.867–3.473	0.120	–	–	–
Drink	2.532	1.452–4.417	0.001	1.184	0.576–2.436	0.646
WBC	1.248	1.051–1.482	0.012	–	–	–
Stroke location						0.010*
Supratentorial	Ref	Ref	0.034	Ref	Ref	
Subtentorial	1.954	1.088–3.509	0.025	2.867	1.357–6.059	0.006
Both	0.706	0.115–4.319	0.707	3.883	0.901–6.670	0.068

RP, C-reactive protein; NLR, Neutrophil to lymphocyte ratio; NIHSS, National Institutes of Health Stroke Scale on admission; SBP, Systolic blood pressure; DBP, Diastolic blood pressure; ALT, Alanine aminotransferase; TC, Total cholesterol; LDL, Low density lipoprotein; WBC, White blood cell.

*P < 0.05.

### Model performance and validation

**FIGURE 3:**
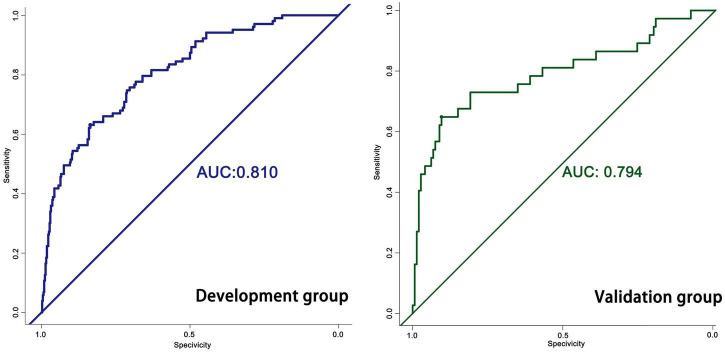
The ROC of the nomogram.

**FIGURE 4:**
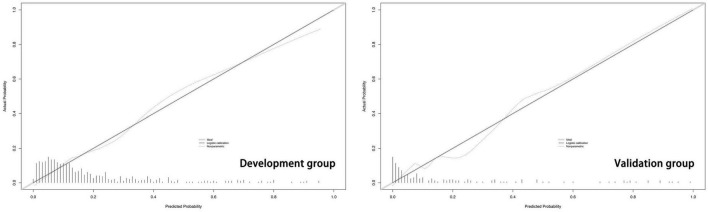
The Calibration plots based on the nomogram.

The predictive model built on the basis of 554 patients in the development group (1 week) was validated for its ability to predict accurately based on the AUC. In the development group, AUC = 0.810, which has good predictive power. An AUC of 0.794 was achieved in 186 externally validated patients, again showing excellent predictive accuracy (Figure 3). The prediction model at one month of onset achieved an AUC of 0.755. Calibration plots are likewise one of the good ways to evaluate the accuracy of the prediction model, as shown in Figure 4, calibration plots show the best agreement between the predicted and observed results.

The second endpoint was the patient’s NGT retention at 1 month of onset. One month after onset is an important time point, the guidelines suggest that percutaneous endoscopic gastrostomy may be a reasonable option if swallowing disorders are likely to persist beyond 1 month (7). We re-analyzed the 5 variables: age, NIHSS, NLR, stroke location and drinking history to assess whether these indicators were equally applicable to predict prognosis at 1 month. After multifactorial logistic regression analysis, patient’s NLR (OR: 1.360, 95% CI: 1.189–1.555, *P* < 0.001), NIHSS (OR: 1.083, 95% CI: 1.018–1.152, P: 0.012) and stroke location (subtentorial vs. suprarenal: OR: 2.867, 95%CI: 1.357–6.059, P: 0.001) retained their effective predictive power. In contrast, age and drinking history lost their previous predictive effects. Accordingly, we redrew the corresponding nomogram at 1 month after onset (Figure 2).

### Patients’ stroke-associated pneumonia at 1 month onset

**FIGURE 5:**
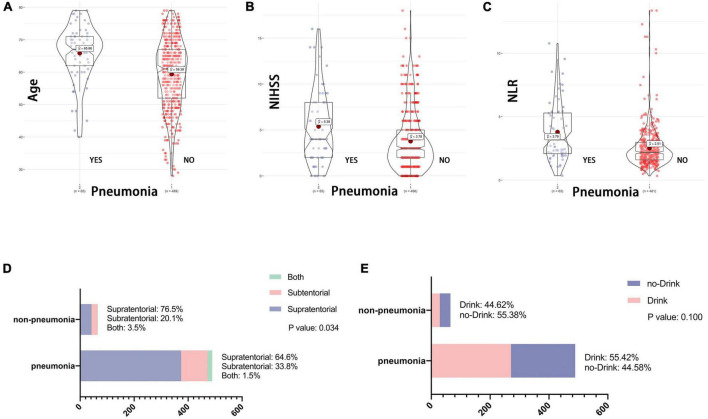
**(A–E)** Association between baseline data and SAP. NIHSS, National Institutes of Health Stroke Scale on admission; NLR, Neutrophil to lymphocyte ratio. One of the endpoint of this study is the presence of stroke associated SAP after AIS. As with the variables included in Figure 1, whether the patient drank at 1 month of follow-up had no effect on the occurrence of SAP. While patient age, NLR, NHISS and stroke location remained statistically different between the two groups.

The third endpoint of the study was the presence of SAP in patients with AIS. Previous studies have shown that patients with post-stroke dysphagia have a threefold higher risk of developing pneumonia than those who do not have dysphagia (2). Therefore, we tried to clarify whether the 5 variables of age, NIHSS, NLR, stroke location and drinking history had an effect on SAP after AIS. As shown in Figure 5, the age (65.86 vs. 59.39), NIHSS (5.38 vs. 3.76), and NLR (3.79 vs. 2.51) were statistically different between the two groups; the stroke location was statistically different by chi-square test, while drinking did not appear to be associated with the development of SAP.

## Discussion

Dysphagia is a common complication after AIS, and we utilized 740 AIS patients to develop a clinical prediction model to predict the likelihood of retaining an NGT within 1 week to guide decision making for NGT feeding in those with dysphagia after AIS. The model incorporates clinical data that are readily available and commonly used in clinical work, making this predictive model easy to use and generalize.

In previous studies, some factors associated with swallowing function in patients with AIS have been explored. Bravata et al. (12) found that using the NIHSS to predict dysphagia had better performance than the nursing admission dysphagia screening tool. Jeyaseelan et al. (13). found the NIHSS to be more sensitive than a 3-sip water test. In Toscano’s study, the NHISS without dysphagia was 4.98 ± 3.50 and with dysphagia was 15.77 ± 7.79 (14). In the study by Henke et al., it was found that the NIHSS cutoff for whether or not post-stroke dysphagia occurred was 4.5, and the mean NIHSS score for all patients was 3 (15). Similar to the study by Henke et al., in our study the mean NIHSS was 3.97 and by calculating the Youden Index we obtained the same cutoff score of 4.5. However, the mean NIHSS for patients with dysphagia in their study was 8 and ours was 5.3. This may be related to selection bias in our study. In addition to this, different definitions of dysphagia criteria may have contributed to this discrepancy. However, the main problem with NIHSS is the lack of sufficient specificity, which correlates with negative predictive values below 40% and positive predictive values of about 90%. This means that patients above the threshold are highly likely to have swallowing problems, but those below the threshold cannot be classified as dysphagia. Therefore, the NIHSS should not be used as the only screening parameter to detect dysphagia, but rather as one of the items to provide clinical decision making. In this study, NIHSS was used as part of the model to participate in the assessment of the likelihood of patients retaining NGT. NIHSS was an independent predictor both at 1 week and at 1 month of onset.

Alcohol consumption is related to dysphagia after AIS. Heavy alcohol consumption in a normal diet reduces the pressure of the lower esophageal sphincter, slowing esophageal motility and gastric emptying, thus promoting acid reflux (16). In our study, although a history of drinking was a risk factor for retention of NGT at 1 week, this association was not present at 1 month.

There are conflicting findings regarding the location of the lesion and dysphagia after AIS. Previous studies had shown that the risk of SAP was associated with brainstem lesions, whereas the relationship between cortical stroke and SAP or dysphagia had not been confirmed (17, 18). In the study by Suntrup et al. (19), there was a significant difference in the percentage of local infarcts in different brain regions between patients presenting and not presenting with specific functional impairment. In particular, lesions of the pre- and post-central gyri, opercular region, supramarginal gyrus and respective subcortical white matter tracts in the right hemisphere are associated with dysphagia, and postcentral lesions are particularly associated with severe swallowing disorders. The swallowing process remains controversial in terms of the degree of lateralization. Several studies have shown that swallowing exhibits asymmetries in the cerebral hemispheres (20, 21). Studies have shown differences in swallowing physiology between right- and left-sided unilateral stroke patients, with pharyngeal damage being more severe in patients with right-hemisphere strokes (22). The only area that predicts severe dysphagia is the right postcentral gyrus, the primary sensory area for swallowing (23). Although these studies were meticulous and interesting, we analyzed the location of the lesion in the modeling group of 554 patients in three aspects of stroke laterality, stroke location and affected arterial territory, and found that stroke laterality (left or right, bilateral) and affected arterial territory (anterior cerebral, middle cerebral, posterior cerebral, vertebral, basilar, or multiple) had no effect on patient retention of NGT. Similar to conventional wisdom, our study found a higher probability of dysphagia in subtentorial lesions than in supratentorial (OR: 1.954, 95%CI: 1.088–3.509, P: 0.025).

In one of our previous studies, NLR had a stronger and statistically significant predictive effect in predicting the prognosis of patients with AIS compared with white blood cells, neutrophils or lymphocytes alone and neutrophils plus lymphocytes (24). As mentioned earlier, among peripheral circulating leukocytes, neutrophils are considered to be an important mediator of ischemic brain injury (25). Experimental evidence suggests that some specific lymphocyte subtypes play a key role in the inflammatory response to chemical injury and are major cerebral protective immunomodulators (26). NLR reflects the balance between neutrophil and lymphocyte levels and their differential immune activity, and NLR has been used as a potential predictor of mortality in patients with AIS (27). This study found that NLR was strongly associated with SAP (3.79 vs. 2.51, *p* < 0.001). This shares a common view with the study by Lan et al. (28). in which NLR was an independent risk factor for AIS-associated infection (OR: 1.16, 95%CI: 1.01–1.33, P: 0.034). Thus, high NLR levels strongly predict a poor prognosis for patients with AIS, both the higher incidence of NGT retention rate and higher SAP were strongly associated with high NLR levels.

The third endpoint of this study was SAP, which we found to be associated with age, NIHSS, NLR, and stroke location. Here we have to think about the question: does the different way of screening for dysphagia have an impact on SAP? In fact, different methods of determining dysphagia will have a significant impact on the occurrence of SAP. An accurate swallowing screening test may be crucial in lowering the incidence of SAP. Indeed, recent studies reported a higher SAP incidence in stroke patients who failed a high-sensitive screening for dysphagia compared to those who passed the screening (4, 29). In addition, the high-sensitivity screening method is more effective in reducing the incidence of SAP than the low-sensitivity screening method. In the study of Jannini et al. found that among patients screened by dysphagia, none of the patients screened by the high-sensitivity method developed SAP compared to 31.82% in the low-sensitivity group (30). Patients screened with low-sensitivity protocols and those who fail to screen are at increased relative risk of SAP and poorer clinical outcomes of stroke due to missed diagnosis of dysphagia.

For SAP, we still have the following concerns. Although SAPs usually occur within the first 7 days after a stroke episode, in half of the cases of SAPs, patients are not affected by dysphagia. In this case, half of the SAPs may appear after 7 days due to immunosuppression and oropharyngeal pathogen colonization associated with tube feeding, especially in the ICU, where the incidence of SAP is as high as 38% (31).

Based on the above, it is likely that a significant proportion of patients who develop SAP are not associated with dysphagia. The body enters a state of immunosuppression to protect the damaged brain tissue, hence attenuating the autoimmune reaction in the brain. However, this stroke-induced immunosuppression predisposes the body to pathogen invasion, leading to stroke-induced immunosuppression syndrome (SIDS) and infection (32, 33). As mentioned in our article, NLR can be used to estimate the degree of immunosuppression that occurs after such strokes, with higher NLR suggesting more pronounced immunosuppression.

Bacterial colonization is especially common in the critically ill for various reasons, including frequently impaired host defenses, the presence of invasive devices that form a nidus for colonization, and the administration of often long-term or repeated courses of antibiotics. Therefore, this fraction of patients with severe SAP may not be caused by dysphagia, but by colonization with colonizing pathogenic bacteria. This part of the SAP patients deserves a deeper investigation in the future.

The present study still has some limitations. First, our study cohort excluded patients who were lost to follow-up. Most patients at day 7 were still hospitalized or able to be followed up on an outpatient basis and could be followed up face-to-face. However, at the 1 month follow-up, most patients were followed up with a telephone questionnaire, which may have caused bias. Many patients with severe stroke were not included in the study, resulting in bias. Second, this study included patients who were hospitalized in large hospitals, and it remains to be studied whether the findings can be generalized downward to lower-level hospitals. Thirdly, we assessed the patient’s degree of dysphagia by V-VST and determined whether the patient needed an indwelling NGT by FOIS. However, this is not the gold standard for assessing dysphagia. Here, instrumental assessment of dysphagia, such as video fluoroscopy or fiberoptic endoscopic evaluation of swallowing, is clearly superior (34). Although instrumental assessment is considered the gold standard, it is also more complex and expensive compared to clinical assessment, making it less widely applicable. Non-instrumental assessment is a shortcoming of this study, while assessment by V-VST has its own advantages. V-VST is a widely validated method for assessing dysphagia that is easy to perform, inexpensive, and has good patient cooperation, making it easy to obtain clinical data. Finally, the present study failed to include hemorrhagic stroke, and subsequent studies may be able to remedy this regret. Factors such as leukoaraiosis, post stroke cognitive impairment, and other factors that may affect dysphagia were also excluded (mainly contrary to the principle of model simplicity). These interesting variables will be studied in a follow-up study.

## Conclusion

We have developed a simple model by utilizing easily accessible clinical variables, which can well assess the possibility of indwelling NGT in AIS patients within 1 week after the onset of AIS, and timely insertion of NGT for patients who may need it to supply adequate nutrition. The model allows for personalized clinical decision making by identifying those individuals who may benefit from NGT feeding.

## Data availability statement

The original contributions presented in this study are included in the article/supplementary material, further inquiries can be directed to the corresponding author/s.

## Ethics statement

The study was approved by the Ethics Committee of the Shanghai Fourth People’s Hospital and complied with the Helsinki Declaration. Written informed consent for participation was not required for this study in accordance with the national legislation and the institutional requirements.

## Author contributions

PZ, Z-LC, and ZS designed the study. WZ and WS interpreted the data. PZ wrote the manuscript. PZ, WZ, and WS performed the statistical analyses. JW, ZZ, CL, and NW screened and extracted the data. ZS and Z-LC supervised the study. All authors have made an intellectual contribution to the manuscript and approved the submission.
